# Vibrational Approach to the Dynamics and Structure of Protein Amyloids

**DOI:** 10.3390/molecules24010186

**Published:** 2019-01-06

**Authors:** Haoqian Li, Richard Lantz, Deguo Du

**Affiliations:** 1 Queen Mary School, Medical School, Nanchang University, Nanchang 330031, China; 6303415034@email.ncu.edu.cn; 2 Department of Chemistry and Biochemistry, Florida Atlantic University, Boca Raton, FL 33431, USA

**Keywords:** protein aggregation, amyloid, oligomers, vibrational spectroscopy, infrared, Raman, isotopic labelling, site-specific probe

## Abstract

Amyloid diseases, including neurodegenerative diseases such as Alzheimer’s and Parkinson’s, are linked to a poorly understood progression of protein misfolding and aggregation events that culminate in tissue-selective deposition and human pathology. Elucidation of the mechanistic details of protein aggregation and the structural features of the aggregates is critical for a comprehensive understanding of the mechanisms of protein oligomerization and fibrillization. Vibrational spectroscopies, such as Fourier transform infrared (FTIR) and Raman, are powerful tools that are sensitive to the secondary structure of proteins and have been widely used to investigate protein misfolding and aggregation. We address the application of the vibrational approaches in recent studies of conformational dynamics and structural characteristics of protein oligomers and amyloid fibrils. In particular, introduction of isotope labelled carbonyl into a peptide backbone, and incorporation of the extrinsic unnatural amino acids with vibrational moieties on the side chain, have greatly expanded the ability of vibrational spectroscopy to obtain site-specific structural and dynamic information. The applications of these methods in recent studies of protein aggregation are also reviewed.

## 1. Introduction

Protein aggregation and amyloid formation has become an important topic in protein biophysics as well as in molecular medicine, in part because amyloidogenesis of a number of aggregation-prone proteins has been recognized as a common pathogenic event in a variety of human diseases including Alzheimer’s, Parkinson’s, Huntington’s, type II diabetes, and others [1,2]. In 1854, Rudolph Virchow coined the term amyloid [3]. Amyloids are protein aggregates composed of insoluble fibers with monomeric strands packed in a cross-β pattern that are stabilized by interstrand interactions such as hydrogen bonding, electrostatic interactions, aromatic interactions (π–π stacking), and hydrophobic interactions [4,5]. The amyloid fibrils themselves, however, are not necessarily the major toxic species. Growing lines of evidence has indicated that the oligomeric intermediates formed during protein fibrillization appear to be more toxic and responsible for neurological damage in some neurodegenerative diseases [6,7]. There is therefore great interest in elucidating the mechanistic details of protein aggregation and the molecular structures formed along the aggregation pathway, for a more comprehensive understanding of the mechanisms of protein oligomerization and fibrillization and development of pharmacological means to ameliorate amyloid toxicity.

**Figure 1:**
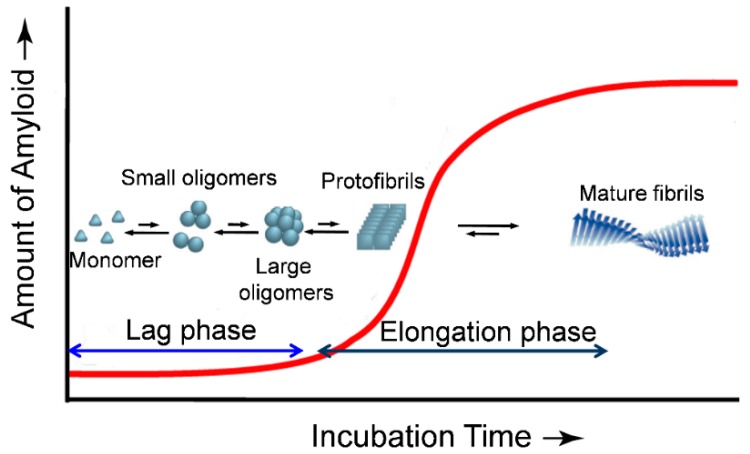
Schematic representation of a typical nucleated polymerization process of protein aggregation. Oligomeric nuclei are formed in the early lag phase stage, being a critical rate limiting step. In an elongation phase, addition of monomers and/or oligomers onto the nucleus allows formation of fibrils which is energetically favorable.

Protein amyloid formation is usually proposed as a nucleated polymerization mechanism in vitro (Figure 1) [8,9,10]. The rate limiting step of this process is the formation of the oligomeric nucleus, the highest energy species on the amyloidogenesis pathway. After the nucleus is formed, additional monomeric peptides or oligomers can be added in a step that is thermodynamically favorable, leading to a fast growth phase to the fibrillar forms (Figure 1). In the past two decades, a combination of widespread techniques has been employed in studying the dynamics of protein amyloidogenesis and the structures of the aggregated species. The cross-β-sheet fibrils can be selectively detected by fluorescent dyes Congo red and thioflavin T (ThT) [11,12]. Techniques such as electron microscopy [13], atomic force microscopy (AFM) [14,15], small-angle X-ray scattering [16], solid/solution state NMR [17,18,19,20], have been used extensively to explore the morphology and structural characteristics of the mature fibrils and oligomeric intermediates. X-ray crystallography, a powerful tool for determining the three-dimensional structure of proteins, has also been employed recently to study amyloid structures formed by relatively short peptides at atomic resolution [21]. However, crystallization of amyloids of larger peptides/proteins is still difficult.

Vibrational spectroscopies, such as infrared (IR) and Raman, are classical methods for investigation of protein structure, dynamics, folding/unfolding, and misfolding [22,23,24]. The molecular vibrations of proteins can be monitored with IR and Raman spectroscopy on the picosecond timescale when used in conjunction with a femtosecond laser [25]. In IR spectroscopy, infrared radiation is used to excite the vibrational modes of the molecule of interest due to a net dipole change [26]. Most molecules, except for homonuclear diatomic molecules, show IR absorption. Technically, Fourier transform infrared (FTIR) spectroscopy is widely used because of its high signal-to-noise ratio, fast data acquisition, and reliable digital subtraction [26,27]. Unlike IR spectroscopy, Raman spectroscopy uses the polarizability of the molecule instead of the net dipole change to observe molecular vibrations. In Raman spectroscopy, the incident radiation excites the sample and causes inelastic scattering where the scattered light is either higher or lower than the incident radiation [28]. Raman spectroscopy shows strong potential for providing noninvasive structural information of proteins. Different techniques, e.g., ultraviolet resonance Raman (UVRR) spectroscopy with higher sensitivity than conventional Raman spectroscopy, have also been successfully applied for studies including protein structures and protein–protein interaction.

Protein misfolding and amyloidogenesis is always associated with changes of secondary structures. The high sensitivity of the vibrational spectroscopies to the secondary structure of proteins makes them particularly valuable for studying the conformational dynamics in protein self-assembly and amyloid formation. The vibrational spectroscopies are well suited for determining the structural features of proteins both in solution and as insoluble aggregates. In additionto the determination of the global structural changes from analysis of the backbone vibration, recent applications of site-specific approaches, such as isotopic labeling of specific amino acid(s) [23], and use of unnatural amino acids with unique mutated side chains (e.g., nitrile, thiocyanate, azide) as vibrational probes [24], has greatly expanded the application of vibrational spectroscopies to explore local dynamics and conformational changes with residue specific resolution. In this paper, we attempt to review some recent research achievements of protein aggregation and amyloidogenesis studies using vibrational approaches including the backbone amide band, isotopic decoupling, and side chain vibrational probes.

## 2. Backbone Vibrational Probe

In the mid-IR region, a polypeptide or protein vibration spectrum that covers the 400 to 4000 cm^−1^ range can be characteristically described as nine frequency regions according to different modes of vibrations [29,30]. Of these, the two most prominent vibrational band regions of proteins are amide I (1610–1700 cm^−1^), arising primarily from the C=O stretching vibration, and amide II (1480–1600 cm^−1^), mainly deriving from the combination of the NH in-plane bend and CN stretching vibration [31]. In particular, the amide I vibration is little affected by the nature of the side chain, and mainly depends on the secondary structure of the backbone. Therefore, the amide I band is the most sensitive spectral region to protein secondary structures of α-helix, β-sheet, turn, and disordered conformations [32,33,34,35]. From the amide I band, one can differentiate between different secondary structures. The absorbance range in the amide I band for a particular secondary structure is summarized in Table 1. For instance, random coil structures show a broad amide I band located at 1640–1650 cm^−1^ [36], while the α-helices and β-sheets have amide I frequencies at approximately 1655 and 1630 cm^−1^, respectively [36,37].

Amyloidogenic peptides, when aggregated, normally exhibit a characteristic cross-β-sheet structure [2,39]. This structure can be probed sensitively by analyzing the amide I band in vibrational spectroscopy. The native β-sheet features an amide I band at 1630–1640 cm^−1^, whereas the amide I band of the aggregated amyloid β-sheets is generally in the range of 1610–1630 cm^−1^, possibly due to being in a more hydrophobic environment and formation of stronger hydrogen bonding [40,41]. More ordered fibers absorb at around 1620 cm^−1^ while more disordered fibers absorb at approximately 1635 cm^−1^ due to stronger coupling [42]. In addition, amyloid fibrils may be composed of parallel or antiparallel β-sheets. In comparison to parallel β-sheets, antiparallel β-sheets exhibit an additional weaker high frequency amide I transition at approximately 1670–1690 cm^−1^ [32,41].

When probing proteins in solution with FTIR, D_2_O is normally used instead of H_2_O due to the strong water bending mode that overlaps the amide I vibrational mode [43]. The spectrum analysis methods like Fourier self-deconvolution, second derivative, and curve-fitting, are commonly used to acquire the secondary structural information. For sampling methods, besides the conventional transmission measurement, attenuated total reflectance (ATR) where the sample is placed on a crystal that has an index of refraction larger than the sample itself, is also commonly used [26]. This technique is advantageous in certain aspects because of the very short pathlength into the sample. It is more amenable to study aqueous samples and the sample preparation is generally much simpler. Moreover, in the past two decades, two-dimensional infrared spectroscopy (2D-IR) has been established as a powerful tool to determine the dynamics of proteins structures in complex systems with high temporal resolution [44,45]. 2D-IR spectroscopy is sensitive to protein secondary structure, vibrational coupling, and solvent exposure based on frequencies and cross-peaks. In comparison to FTIR, 2D-IR allows the resolution of cross-peaks, which reveal coupling between different vibrational modes. Unlike FTIR spectroscopy, 2D-IR signals scale with the fourth power of the transition dipole moment, resulting in enhanced resolution of peaks in the spectra [46]. In Raman spectroscopy, the H_2_O bending vibration mode that obscures the amide I band in IR spectroscopy has a low intensity, obviating the need to use D_2_O in Raman spectroscopy measurement, and reduces the errors inherent in large solvent background subtractions.

With these vibrational techniques, one can study structure characteristics of protein aggregates, and probe the conformational dynamics in real-time along the aggregation process. Discussed below, are examples of some recent studies in the structural properties and kinetics of aggregation of a number of amyloidogenic peptides/proteins using the backbone vibrational probe.

### 2.1. Amyloid-β

**Figure 2:**
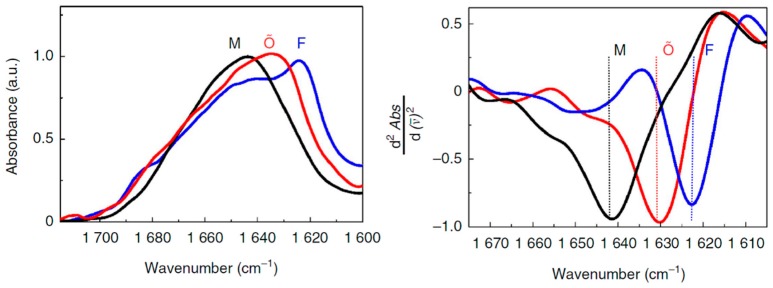
**Left panel**: normalized FTIR spectra of Aβ_1–40_ monomers (M) in 10 mM HEPES/D_2_O pD11, fibrils (F) after 24 h incubation in 10 mM HEPES pD7.4, and Cu(II)-induced oligomers (Aβ_1–40_-Cu(II)Õ) after 24 h incubation at 37 °C, pD7.4 [51]. **Right panel**: second derivatives of the FTIR spectra. Reprinted with permission.

Alzheimer’s disease (AD) is the most common neurodegenerative disorder. One of the hallmarks of AD is the formation of extracellular deposits of amyloid-β (Aβ) in the brain. Aβ peptides are cleaved from the amyloid-β precursor protein (APP) and aggregate to form oligomers and then ultimately to insoluble fibrils that are made up of β-sheets [47]. The majority of the secreted Aβ peptides are Aβ_1–40_ and Aβ_1–42_, which start at Asp1 and end at either Val40 or Ala42, respectively. Compelling genetic, biochemical and pathological evidence indicates that the etiology of AD is mechanistically linked to the production and aggregation of Aβ [48,49]. A growing body of recent studies has suggested that the oligomeric, diffusible assemblies of Aβ peptides formed in the early stages of aggregation, rather than the mature amyloid fibrils, may be the primary neurotoxic species in AD. The mechanisms of the conformational changes in the formation of oligomers and fibrils of Aβ therefore have been the subject of numerous in vitro studies. FTIR spectroscopy has been extensively used to study the conformational dynamics and the alignment of β-strands in the formation of Aβ oligomers and amyloid fibrils in vitro. In an ATR-FTIR study, Sarroukh et al. reported that conversion of Aβ_1–40_ oligomers into fibrils results from a transition from antiparallel to parallel β-sheet, by observing a progressive vanishing of a peak at 1695 cm^−1^ which is typical of an antiparallel arrangement of the β-strands [50]. The presence of an amide I band at ~1630 cm^−1^ in the intermediates suggests the formation of β-sheet structure in oligomers. The signature of this low frequency β-sheet band in Aβ_1–40_ oligomers was also reported in another recent study of Klementieva et al. [51]. A broad band centered around 1640 cm^−1^ (black, M) corresponds to unstructured Aβ_1–40_ monomers (Figure 2). A band centered at around 1623 cm^−1^ (blue, F) suggests the existence of fibrillar β-structures of amyloids. Importantly, as shown in Figure 2, the Aβ_1–40_ oligomers prepared in the presence of ions of Cu(II) show a peak at about 1630 cm^−1^ (red, Õ), which indicates the presence of β-sheets, although the electron microscopy, small angle X-ray scattering and ThT spectroscopy data support the non-fibrillar nature of these Aβ_40_ oligomers. The secondary structural features of the oligomers can therefore be discriminated from that of the amyloid fibrils. The spectroscopic signatures of the stable amyloid fibrils have been also distinguished from oligomeric intermediates using more sensitive 2D-IR spectroscopy. A unique transition at 1610 cm^−1^ is observed in the 2D-IR spectra of the mature fibrils of both Aβ_1–40_ and Aβ_1–42_ [52]. This band does not appear in other Aβ aggregates including β-sheet-structure-like oligomers, and is not seen in linear IR spectroscopy because it is occluded by the broad band at ~1625 cm^−1^. Interestingly, the 1610 cm^−1^ band still exists even when the Aβ aggregates are solubilized in sodium dodecyl sulfate (SDS), indicating that the 1610 cm^−1^ transition corresponds to highly stable amyloid species that are not disaggregated by SDS.

IR spectroscopy has also been applied to illuminate the secondary structure characteristics of Aβ in complex biological conditions. In a study of Klementieva et al., the FTIR spectra maps were recorded in brain sections of both AD transgenic Tg19959 mice and wild-type mice to identify the increase of β-sheet contents in AD mice over time [51]. Similarly, FTIR spectroscopy was used to compare the structural characteristics of the Aβ aggregates formed in vivo to that formed by chemically induced aggregation ex vivo [53]. Recently, Wiltfang and co-workers developed an immune-infrared sensor to measure the Aβ peptide secondary structure distribution in cerebrospinal fluid (CSF) and blood plasma [54]. The amide I band frequency downshifts to a β-sheet secondary structure in Dementia Alzheimer type patients, and the discrimination between the Dementia Alzheimer type patients and the disease control patients reaches an accuracy of 90% for CSF. This kind of method holds a promise for simple, robust, and label-free diagnosis of this devastating disease.

### 2.2. Islet Amyloid Polypeptide

Human islet amyloid polypeptide (IAPP) is a 37-residue peptide hormone secreted by pancreatic β-cells that acts with insulin as a regulator of glucose homeostasis. IAPP is a natively disordered and highly amyloidogenic peptide that easily self-assembles into amyloid fibrils via a multistep process. The aggregation and amyloid formation of IAPP is strongly associated with β-cell degeneration in type II diabetes [55,56]. Similar to Aβ, it has been proposed that the oligomers of IAPP might be the major toxic species that lead to β-cell death [57]. The structural characteristics of the IAPP oligomers have also been investigated by vibrational spectroscopy. Rawat and co-workers used both FTIR and Raman spectroscopy to investigate the conformation of the peptide chain in the different aggregation states of IAPP [58]. Both FTIR and Raman spectra of the IAPP oligomers suggest a predominantly α-helical conformation (together with significant β-sheet content) of the peptide chain in the oligomeric state, while in fibrils the peptide is predominantly in a β-sheet conformation. This is consistent with circular dichroism (CD) studies of IAPP revealing the formation of α-helical states in IAPP aggregation [59]. 2D-IR spectroscopy is also applied to discriminate different secondary structural elements during amyloid formation without the need of deconvolution of the spectra [60]. Abedini et al. performed a 2D-IR study on IAPP oligomers to define the structural properties of the toxic IAPP intermediates [61]. Their results indicated that the more toxic oligomers contain flexible and low order structure with modest overall β-sheet and α-helical content.

The structural features of the mature fibrils of IAPP and their different isoforms have also been studied by vibrational spectroscopy of the backbone. Zanni and co-workers used 2D-IR to investigate the structural diversity of the amyloid fibrils of human IAPP [62]. The presence of an inhomogeneously broadened β-sheet peak and strong coupling to random coil conformations reveals a large structural distribution of the fibrils. In a later publication, they analyzed the secondary structural properties of human IAPP and rat IAPP in solution, membrane, or micelle bound forms by measuring the transition dipole strengths of the samples using both 1D and 2D-IR spectroscopy [63]. The amide I band has also been used to evaluate the function of inhibitors in blocking IAPP aggregation [64,65,66,67]. The inhibition activity of small compounds, such as rhodamine derivatives and red wine compound resveratrol, on IAPP aggregation was confirmed by monitoring the amide I band change using ATR-FTIR [64,65]. Using similar methods, Sellin et al. reported that a non-amyloidogenic human IAPP analog and a hexapeptide have strong inhibitory effects on IAPP fibrillization at the membrane interface, suggesting that these peptides may be able to suppress pathogenic self-association of IAPP also in vivo [66].

### 2.3. α-Synuclein

Parkinson’s disease (PD) is the second most common neurodegenerative disorder characterized by formation of cytosolic inclusions known as Lewy bodies in the neurons of the brain [68]. α-Synuclein, a 140-residue presynaptic protein, has been shown to be a major fibrillar component of Lewy bodies, and the mutations to the α-synuclein gene cause early onset of PD [69,70,71], implicating the aggregation of α-synuclein as a key step in the etiology of PD. Along with Aβ and IAPP, it has been suggested that the oligomeric species of α-synuclein are more toxic than mature fibrils to cause neuronal death [72]. While being natively disordered under neutral pH, α-synuclein is transformed into a partially folded conformation with a significant amount of β-structure at acidic pH, evidenced by appearance of a new band in the vicinity of 1626 cm^−1^ [73]. The amide I absorption of α-synuclein in the oligomer-forming conditions exhibits a band at 1625 cm^−1^ along with a prominent shoulder at 1695 cm^−1^, indicating the components of antiparallel β-sheet structure in oligomers; whereas the amyloid fibrils displayed the typical parallel β-sheet features characterized by a maximum band at 1628 cm^−1^ [74]. However, a deconvolution analysis of the FTIR spectra of α-synuclein and three variants reveals the antiparallel β-sheet structure in α-synuclein fibrils [75]. These contradictory results may imply the sensitivity of the structure of α-synuclein aggregates to environments. Indeed, a recent study of Roeters et al. using a combination of FTIR, 2D-IR and AFM suggested that α-synuclein fibrils formed in low-salt buffers are composed of loosely packed parallel β-sheet structure with extended conformation, while the fibrils formed in high-salt buffers mainly adopt a more tightly-packed, antiparallel intramolecular conformation [76].

In addition to IR, Raman spectroscopy has also been applied to characterize the conformation of the natively unfolded α-synuclein in various solvents before fibrillization using Raman amide I and III (which is also sensitive to secondary structure) bands [77,78,79]. The amide III band is a combination of CN and NH stretching in the region of 1200–1340 cm^−1^ [80]. This band is also known to be structurally sensitive owing to its dependence on the psi and phi dihedral angles [80,81,82]. The Raman studies on monomeric α-synuclein, conducted by Anderson group, showed that the secondary structure is largely α-helical in hexafluoro-2-propanol (HFIP) and SDS, and predominantly β-sheet in 25% methanol in H_2_O [77]. The characterization of the secondary structure of α-synuclein oligomers by analyzing the Raman amide I band profiles showed that the spherical oligomers have a significant amount of α-helical structure [78], which decreases in protofilaments and filaments accompanied by the increase of the β-sheet structure. Upon filament formation, the Raman amide I band narrows dramatically accompanied by a red shift of the peak maximum, consistent with a progressive increase in β-sheet structure and the formation of more ordered aggregates.

### 2.4. Examples of Other Disease-Associated Proteins and Model Peptides

In addition to the aforementioned amyloidogenic proteins, the backbone amide I spectra have been widely used in aggregation studies of other disease-associated amyloidogenic proteins, e.g., crystallin [83], prion [84,85], polyglutamine (polyQ) [86], and model peptides [87,88]. For instance, deposits of aggregated crystallin on the lens of the eye cause blurred vision or blindness in cataracts. The antiparallel β-sheet structure was identified by FTIR when γD-crystallin was incubated at acidic pH mimicking the lysosome compartments of the eye [83]. In a recent study, although not observable in TEM imaging because of the small size, Zhang et al. was able to identify the formation of the ordered β-sheet amyloid structure of γD-crystallin in UV-induced cataracts of porcine lenses, owing to the enhanced sensitivity of 2D-IR to amyloid β-sheet secondary structure by non-linear scaling of 2D-IR intensities and cross peaks [89]. Such pioneering work expands the ability of application of 2D-IR spectroscopy in more complex tissues studies. Taken together, the vibrational spectrum arising from protein backbone have been continuously employed as a versatile and convenient method for identifying the secondary structural features and monitoring the conformational dynamics of both the metastable oligomers and the mature fibrils of amyloidogenic proteins with distinct primary sequences. Differentiating the structural characteristics of oligomers from fibrils may be crucial for understanding the strong cytotoxicity of the oligomeric species.

## 3. Isotopic Labeling Probe

The amide I band of peptides and proteins is generally applied as a global probe of the assembled secondary structures, but it is hard to be assigned to specific residues or local regions of the protein. To overcome this limit, development of site-specific isotopic labelling method combined with vibrational spectroscopy, has greatly advanced the ability of vibrational approaches to provide information of protein structure and dynamics with higher resolution [90,91,92]. Isotopic editing allows one to replace residue(s) of interest with analogues bearing an isotope-labeled ^13^C=^16^O or ^13^C=^18^O carboxylic group in a noninvasive manner [93,94,95]. Labelling with ^13^C=^16^O induces ~40 cm^−1^ red shift of the amide I frequency, and labeling with ^13^C=^18^O induces a more significant red shift of ~65 cm^−1^ [23,24,96], allowing the frequency of the labeled residues resolved from the bulk unlabeled amide I frequencies. Because of this great advantage, there is already a wealth of studies to identify the local conformational dynamics in protein folding using isotope-edited vibrational spectroscopy [97,98,99,100,101]. Here, we address some of the recent accomplishments of this technique in exploring local structural characteristics along the formation of oligomers and amyloid fibrils of amyloidogenic proteins, e.g., Aβ and IAPP.

### 3.1. Amyloid-β

Although the parallel β-sheet structure has been well resolved in amyloid structures of Aβ_1–40_ and Aβ_1–42_, a shorter Aβ fragment, Aβ_16–22_, forms aggregates with antiparallel in register β-sheet with the central residue (Phe19) in alignment across all the strands, validated by the FTIR study of a series of Aβ_16–22_ mutants with a single ^13^C=^16^O label or two residues labeled simultaneously [102]. This result is consistent with a following study of Shanmugam et al. through isotope-assisted vibrational circular dichroism [103]. In a recent study, Hochstrasser and co-workers incorporated ^13^C=^18^O isotopic substitution to five residues of Aβ_1–40_, respectively, and investigated the 2D-IR spectra of the isotopically diluted amyloid fibrils of Aβ_1–40_ [104]. Their results identified 1D excitation formed by the isotope dilution of parallel in-register β-sheets. The variability of the spectral shifts of the amide I modes for different residues further reveals a structural and/or environmental heterogeneity of the fibrils. To elucidate the structure features of monomeric Aβ, Zhuang et al. investigated the spectral characteristics of Aβ_1–42_ conformers by simulating the 2D-IR spectra of Aβ with ^13^C=^18^O labels at 31–34 and the 38–41 regions [105], which are basically random coil in the monomeric state. In addition, the residue-specific binding of the copper ion with the N-terminal region of Aβ at various pH conditions was also identified by ATR-FTIR spectroscopy in combination with isotopic labeling of the amino acids involved in the coordination sphere [106].

Isotopic labelling was also applied to investigate Aβ aggregation under different environments. There is accumulating evidence suggesting that membranes play a crucial role in amyloidogenesis of Aβ under physiological conditions. Ganglioside GM1 is abundant in the brain and has multiple roles in the function of the brain. It has been demonstrated that GM1 can act as a seed for Aβ growth and the fibrils formed are more toxic than fibrils grown in aqueous solution [107]. Okada and co-workers conducted ^13^C labelled FTIR to distinguish the structural characteristics of Aβ_1–40_ fibrils formed in aqueous solution or on GM1 clusters, and their results suggested a novel mixed parallel and antiparallel β-sheet structure formed on GM1 clusters with almost the entire sequence of Aβ included in the β-sheet [108]. Their findings also showed that GM1 bound fibrils formed faster, had a flat tape like structure, and exhibited stronger hydrogen bonding than fibrils grown in aqueous solution [108,109]. Isotopic labeling was also applied to reveal the vibrational frequency dynamics of 18 individual residues between Val12 and Val39 of Aβ_1–40_ fibrils, to identify the presence of water at specific locations in the fibril [110]. There was water trapped within the fibrils even after years of incubation [111]. This observation was later supported by molecular simulations on Aβ_1–40_ in two different protonation states (one ionized and one neutral) [112]. The authors compared the simulation results to the isotope-edited 2D-IR experiment and concluded that water molecules trapped inside the fibrils play a major role in the frequency fluctuation.

### 3.2. Islet Amyloid Polypeptide

**Figure 3:**
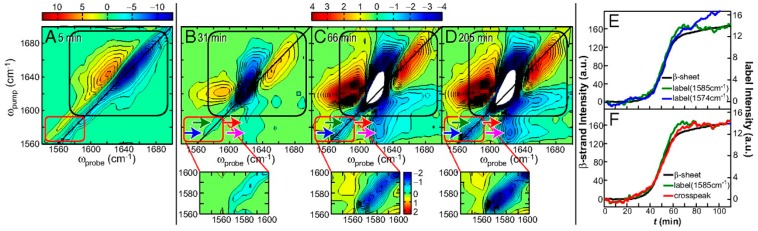
2D-IR spectra and kinetics curves of human IAPP with ^13^C=^18^O labeled at the Ala25 residue [113]. (**A**) The first 2D-IR spectrum at t = 5 min. (**B**–**D**) Difference 2D-IR spectra at t = 31, 66, and 205 min, calculated by subtracting the t = 5 min spectrum. Black boxes surround the spectral features of the unlabeled portion of the peptide, whereas red boxes enclose the diagonal peaks of the isotope labeled Ala25. Blue and green arrows highlight the 2 labeled features, whereas magenta and red arrows point to the cross-peak between the ^13^C=^18^O Ala25 and the unlabeled β-sheet. (**E**) Kinetics of the diagonal peaks of the unlabeled β-sheet at 1617 cm^−1^ and the 2 label features (blue and green arrows). (**F**) Comparison of the kinetics of the cross-peak and the diagonal peaks. Reprinted with permission.

Isotopic labeling has also been used to probe the aggregated structure of human IAPP. In one example, ^13^C=^18^O isotopic labels were put in seven positions along the human IAPP sequence. The results, in combination with the experiments and simulations, showed that the amide I frequency corresponding to a β-sheet is sensitive to the length of β-sheet and the position of isotopic labels [46]. A high frequency at ~1665 cm^−1^, assigned to coupling in the turn region, was also observed and sensitive to the label position within the turn. In a follow-up study, Zanni and co-workers monitored the kinetics of IAPP aggregation at six isotopically labeled sites [113]. For example, as depicted in Figure 3, for the IAPP mutant with ^13^C=^18^O label at Ala25, the 2D-IR spectrum shows that the isotope-labeled features appear near 1580 cm^−1^. The difference spectra highlight that concurrent with the growth of the β-strand, 2 isotope-labeled features appear at 1574 and 1585 cm^−1^. The growth of the large cross-peaks between the isotope labels and the unlabeled β-strand peak at 1617 cm^−1^ indicates that Ala25 is strongly coupled to the β-sheets. Interestingly, the kinetic traces of the intensity of the peaks for the unlabeled β-strand, Ala25, and the cross-peak as a function of time are virtually identical, indicating a direct assembly of the Ala25 residue into a β-strand structure when it becomes part of the ordered fibril structure. A detailed multistep aggregation pathway of IAPP starting with formation of nucleus at the loop region was proposed accordingly from this thorough residue-specific amyloidogenesis study [113]. Since parallel β-sheet formation seems to be significant in the formation of IAPP amyloids, the vibrational coupling was further systematically investigated by six combinations of doubly ^13^C=^18^O isotopic labeling in a synthetic cyclic peptide containing parallel β-sheet structure to establish calculated and experimentally verified coupling models that link spectra to structure [114]. Furthermore, the disruptive effect of a post-translational modification, deamidation of asparagine and glutamine, on N-and C-terminal β-sheet in IAPP amyloid structure was also elucidated by Zanni group using isotope-edited 2D-IR [115].

The structural properties of the transiently populated oligomers of IAPP have also been probed using isotopic labels. By using 2D-IR coupled with isotopic ^13^C=^18^O labelling, Buchanan et al. discovered that an oligomeric intermediate containing a parallel β-sheet structure extending over a hydrophobic fragment 23–27 (FGAIL) forms in the lag phase of IAPP amyloid formation [116]. This local hydrophobic region initially starts out as a random coil structure and evolves into β-sheet oligomers, then is disrupted and forms partially disordered loop during fibril formation [117]. A further study by isotopically labeling two neighboring amino acids in IAPP showed that up to 38% of monomeric IAPP peptides in aqueous solution adopt an α-helical structure at the L12A13 region, but not at the L16V17 residual region [118]. The N-terminal helices of IAPP monomers may help seed IAPP oligomer formation by stabilizing small β-sheet oligomers.

In addition, isotopic labelling has been applied to identify the structural information of amyloid-inhibitor complexes [116,119]. Rat IAPP has been found to be a modest inhibitor of human IAPP aggregation [120]. The residue-specific structural information of human IAPP-rat IAPP complex was studied using isotope-edited 2D-IR spectroscopy [119]. The results showed that rat IAPP inhibited the N-terminal β-sheet instead of the hypothesized C-terminal β-sheet of the human IAPP. Interestingly, it was found that the rat IAPP formed its own β-sheet which was not previously recognized. This kind of study provides residue-specific details of the inhibition mechanism, and may illuminate the development of novel means for blocking IAPP aggregation via targeting the key local residues/regions involved in the oligomer and amyloid formation.

### 3.3. Examples of Other Disease-Associated Proteins and Model Peptides

The local mechanistic details of aggregation of γD-crystallin were thoroughly investigated by the Zanni group using isotopic labelling 2D-IR [121,122,123,124]. The ^13^C labeled N-terminal or C-terminal domains of γD-crystallin were expressed to prepare the full-length protein via protein ligation, and the 2D-IR studies demonstrated that the C-terminal domain is the fibril nucleation site and forms amyloid β-sheets, whereas the N-terminal domain is largely disordered while lying in close proximity to the β-sheets [121]. Misfolding and conformational conversion of prion protein (PrP) into β-sheet rich aggregates is associated with a group of fatal neurodegenerative disorders also known as prion diseases. The structure and mechanism of the aggregation of the prion peptide H1 (residues 109–122 of the prion protein) was addressed using isotope-edited FTIR [125,126]. The residue-level alignment of a kinetically trapped intermediate with antiparallel β-sheet and the subsequent rearrangement of the structure into a more stable conformation with nonexponential local kinetics were reported [127]. In addition, the β-sheet packing pattern of the oligomers and fibrils of the model polyglutamic acids was studied by Keiderling and co-workers using the ^13^C labelled ATR-FTIR and vibrational CD (VCD) spectra [128]. They deduced that the oligomers are made up of antiparallel β-sheets that are stacked and twisted. The amyloid fibril structure and aggregation kinetics of a model polyQ peptide was also investigated by Buchanan et al. via studying fibril formation of a mixture of ^12^C and ^13^C protein mixtures [129]. Investigation of the structural ordering in aggregation of a synthetic hexapeptide AcWL_5_ with a single isotopic label in the presence of lipid bilayer via 2D-IR provides novel insight into the residue-level structural ordering of the aggregated peptide in membrane environments [130,131]. Elucidation of the mechanistic roles of the key residues and local regions in protein aggregation significantly facilitates a comprehensive understanding of the mechanisms of protein amyloidogenesis, and will illuminate future simulation approaches to address protein aggregation process at an atomic-level.

## 4. Side Chain Vibrational Probe

Albeit the power of the isotopic labelling in studying high resolution dynamics of proteins, the method has its own limitations. The natural abundance of ^13^C of ~1% can cause a significant fraction of ^13^C=^16^O amide I modes at random positions. In addition, isotope labeled amide I vibrations are normally located at the 1550–1600 cm^−1^ region, where it is often congested with side-chain vibrations from some of the amino acids [132]. In the past decade, alternative strategies of development and application of extrinsic vibrational probes, many of which are unnatural amino acids with vibrational moieties at the side-chain, have also received great interest for improving the structural resolution of vibrational spectroscopy at a site-specific level. Many of the suitable labels show a vibrational spectrum window at a much less congested region, e.g., between 1900 and 2900 cm^−1^ [133]. These probes can sensitively detect side chain environmental changes and the interactions that don’t necessarily involve the backbone. In order to be useful for monitoring the local dynamics, the probe should be sensitive to the local environment with a relatively intense absorption in a frequency region that is not overcrowded with other vibrations, and importantly, it should cause minimal structural perturbation of the target molecules [134]. Up to now, a large group of useful vibrational probes have been developed and successfully employed in studying monomeric protein structure and dynamics, which are summarized in a number of reviews [24,135,136]. Here, we will more specifically focus on the application of some vibrational probes including azide, nitrile, and ester carbonyl, in studying local dynamics and environmental information in protein amyloidogenesis. There is no doubt that the application of side chain vibrational probes in protein aggregation studies is not restricted to the ones discussed below. It is expected that other extrinsic vibrational moieties, e.g., thiocyanate [137,138], carbon-deuterium (C-D) [139,140,141], may also be employed as valuable local probes in future protein misfolding and aggregation studies.

### 4.1. Azide Probe

Azide-functionalized amino acids, such as β-azidoalanine, azidohomoalanine, and *para*-azidophenylalaline normally show an asymmetric stretch vibration in the region of ~2100 cm^−1^ [133,142]. This region is uncongested because few functional groups present in proteins absorb in this region of the IR spectrum. The size of the azide moiety is relatively small, so the presence of this group on amino acid side chain is unlikely to perturb aggregation significantly. These azide labelled amino acids also have moderately strong extinction coefficients of around 300–500 M^−1^ cm^−1^ which makes them useful for measurement at lower concentrations [133,142]. Although the presence of a Fermi resonance between a combinational band and the N3 asymmetric stretching band may complicate the band profile of the vibration [143], these vibrational probes have been proven to be useful to examine local dynamics and folding of proteins [144,145,146,147,148]. The application of the azide probe to study protein aggregation is still in its early stages. Cho and co-workers incorporated an unnatural β-azidoalanine in Aβ_16–22_ (a peptide with residues 16–22 of the full-length Aβ) to replace Ala, for studying site-specific information of the local electrostatic environments in the aggregates [149]. They found that the azido peak frequency in the aggregates is the same to that in DMSO, suggesting that the vibrational probe is surrounded by a hydrophobic environment in the aggregated state of the peptide, instead of exposed to water. The study suggests that the azide probe can provide sufficient sensitivity with strong intensity for monitoring the local environmental change along the aggregation pathway. Future studies of using these tools will be expected for elucidating high resolution dynamic information in protein aggregation.

### 4.2. Nitrile Probe

Nitrile groups are also excellent vibrational probes of protein structure and dynamics. Like the azide probe, the vibrational frequency of the nitrile groups is also in a relatively clear region of 2100–2400 cm^−1^ [150], and highly sensitive to the local environment. The extinction coefficient varies from 120 M^−1^ cm^−1^ to 800 M^−1^ cm^−1^ and is large enough to make it a viable vibrational probe [134,137,151,152]. There are several nitrile labelled amino acids, e.g., 5-cyanotryptophan, β-cyanoalanine, and *p*-cyanophenylalanine that have been developed in recent years [134,151,153]. Among these, *p*-cyanophenylalanine (Phe_CN_) has received a great deal of attention as a useful spectroscopic reporter of protein structure and dynamics [154,155,156,157,158,159]. Phe_CN_ is a good fluorophore, and the fluorescence quantum yield decreases upon dehydration [154,155,156,157]. Furthermore, the CN stretching frequency of Phe_CN_ at 2220–2250 cm^−1^ is sensitive to the electric field in its environment [160,161] and solvent polarity [134,135,162], making it a suitable vibrational probe to the local environment. For example, in H_2_O, the CN stretching vibrational band of Phe_CN_ is centered at ~2237 cm^−1^, whereas this band shifts to ~2229 cm^−1^ in a less polar tetrahydrofuran (THF) solvent [134,163]. Moreover, substitution of Phe/Tyr with Phe_CN_ introduces little structural perturbation because of their structural similarity. The Phe_CN_ residue can be incorporated into the sequence by either chemical peptide synthesis, or site-specific genetic incorporation methods via evolved aminoacyl-tRNA synthetase/tRNA pair that can specifically target on the unnatural amino acid [134,164,165]. Phe_CN_ has been used widely as a site-specific spectroscopic reporter of protein conformational changes, folding, and hydration states of proteins [156,165,166,167,168,169].

**Figure 4:**
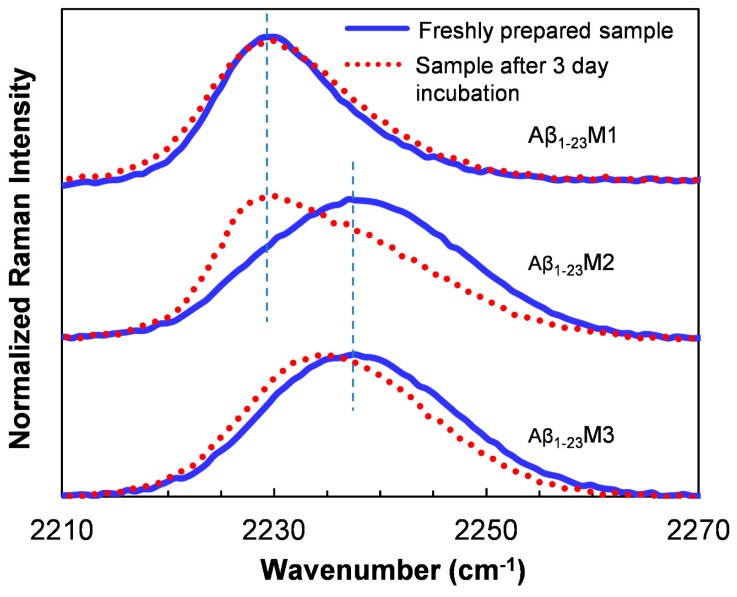
Raman spectra of three Aβ_1–23_ mutants before and after being incubated for 3 day for aggregation [172]. In the mutants, the Phe_CN_ residue was used to replace Tyr10 (Aβ_1–23_M1), Phe19 (Aβ_1–23_M2), and Phe20 (Aβ_1–23_M3), respectively. The vertical dashed lines indicate Raman wavenumbers at 2229 and 2237 cm^−1^, respectively. Reprinted with permission.

Recently, Phe_CN_ has been used to monitor local structural characteristics of amyloid fibrils [170,171,172]. Inouye et al. substituted Phe19 and Phe20 with Phe_CN_ to probe the local hydration states in Aβ_16–22_ [171]. In addition, Raleigh and co-workers replaced the aromatic residues in IAPP with Phe_CN_ to distinguish their local environments upon aggregation [170]. They concluded that the Phe_CN_ residues at 23 and 37 are in a more hydrophobic environment (CN vibration at ~2229 cm^−1^) while residue 15 is in a more water exposed environment (CN vibration at ~2233 cm^−1^) [163]. In a study of the aggregation of an Aβ_1–23_ peptide, Liu and co-workers substituted the aromatic amino acids in the sequence to Phe_CN_ and studied the Raman spectra of CN upon aggregation to reveal distinct local environmental changes of specific residues during the aggregation [172]. For instance, after amyloid formation, the peak of the CN stretching band of the Phe_CN_19 residue shows a significant red shift from 2237 cm^−1^ to 2229 cm^−1^ (Aβ_1–23_M2, Figure 4), indicating a more hydrophobic and less solvent accessible environment for the CN probe in fibrillar structure [163]; whereas the CN band of the Phe_CN_20 residue only shows a red shift of only 2 cm^−1^ of the peak upon aggregation (Aβ_1–23_M3, Figure 4), suggesting a much more polar local environment of this residue in fibrils. In a similar study of the Aβ_1–40_, the CN stretching vibration band in the Raman spectra of all the mutants is centered at approximately 2229 cm^−1^ after aggregation, suggesting a dehydrated and hydrophobic local environment at the mutating positions in the amyloids [173].

### 4.3. Ester Carbonyl Probe

The stretching vibration of the ester carbonyl side chain of a number of unnatural amino acids has also been employed to examine the local electrostatic environment during protein folding and aggregation. A computational study by Choi and Cho predicted that the stretching mode of such a carbonyl group is not only localized, but its frequency also varies linearly with the electrostatic field for both hydrogen bonding and non-hydrogen-bonding environments [174]. The ester carbonyl group absorbs in a spectral region (1700–1800 cm^−1^). Recently, the ester carbonyl stretching vibration of unnatural amino acids, e.g., l-aspartic acid 4-methyl ester, and l-glutamic acid 5-methyl ester, has been reported to be a sensitive probe for local electrostatic and/or hydration environment in a site-specific manner [175]. Gai and co-workers made this probe more applicable to amyloid aggregation by demonstrating that l-aspartic acid 4-methyl ester is sensitive to the hydration and hydrogen bonding of the residue and the local electrostatic field in aggregation of a model peptide of Aβ_16–22_ derivative [175]. The same probe was also introduced to another Aβ_16–22_ peptide derivative to discriminate the hydration status of local residues for dry fibrils and fibrils in aqueous solution by measuring the ester carbonyl stretching vibration [176]. Similarly, a methyl ester group was also introduced to the side chain of the cysteine residue of amyloidogenic peptides via cysteine alkylation, to successfully probe the local hydration state and the structural integrity of the amyloid fibrils [177]. These studies highlight the potential utility of the ester carbonyl stretching vibration as a convenient means for structural determination of amyloids fibrils and local environmental information along the aggregation pathway. Future efforts to further identify and develop novel side chain groups that fulfill the requirements of useful vibrational probes will continue to expand the application of the vibrational spectroscopy in studying protein aggregation with enhanced structural and spatial resolution. It is worth noting that the potential perturbation of the introduced probes on the aggregation properties of the target proteins should always be taken into consideration in such studies.

## 5. Conclusions and Outlook

In summary, being one of the most widely used techniques in the analysis of protein secondary structure, vibrational spectroscopy is still employed as a convenient and powerful means in dissecting structural dynamics and conformational changes of peptides and proteins. The versatility of the vibrational approach and the wide range of time scales makes it particularly valuable in the analysis and understanding of the complex aggregation problem of proteins, complementing other traditional techniques. Furthermore, application of backbone isotopic labelling or side chain vibrational probes, combined with the traditional vibrational technique, allows dissecting the structural and dynamic information of protein oligomer and fibril formation at a site-specific level. We anticipate that the application of these techniques will improve the elucidation of the protein aggregation process in more complex environments, leading to a better understanding of protein aggregation mechanism in vivo. Moreover, in the future, it is expected that the ongoing advances in vibrational spectroscopy, in combination with other experimental and computational methods, will hold promise for facilitating the development of novel strategies for diagnosis and therapeutic treatment of amyloid diseases.

## Figures and Tables

**Table 1:** Assignment of amide I band positions to secondary structure [30].

Secondary Structure	Band Position in H_2_O/cm^−1^		Band Position in D_2_O/cm^−1^	
	Average	Extremes	Average	Extremes
α-helix	1654	1648–1657	1652	1642–1660
β-sheet	1633	1623–1641	1630	1615–1638
β-sheet	1684	1674–1695	1679	1672–1694
Turns	1672	1662–1686	1671	1653–1691
Disordered	1654	1642–1657	1645	1639–1654

Based on the experimental data and assignments of various authors collected and evaluated by Goormaghtigh et al. [38].
